# Antibacterial Effects of Essential Oils on *P. aeruginosa*, Methicillin-Resistant *S. aureus*, and *Staphylococcus* spp. Isolated from Dog Wounds

**DOI:** 10.3390/ph17111494

**Published:** 2024-11-07

**Authors:** Merve Gizem Sezener Kabay, Sinem Inal, Sedat Gökmen, Volkan Enes Ergüden, Arzu Fındık, Tolga Güvenç, Hülya Kayhan, Dilek Güvenç

**Affiliations:** 1 Department of Microbiology, Faculty of Veterinary Medicine, University of Ondokuz Mayıs, Atakum 55270, Samsun, Turkey; gizem.sezener@omu.edu.tr (M.G.S.K.); ergudenv@gmail.com (V.E.E.); afindik@omu.edu.tr (A.F.); 2 Department of Pathology, Faculty of Veterinary Medicine, University of Ondokuz Mayıs, Atakum 55270, Samsun, Turkey; sinem.inal@omu.edu.tr (S.I.); tguvenc@omu.edu.tr (T.G.); 3 Department of Pharmacology and Toxicology, Faculty of Veterinary Medicine, University of Kastamonu, Kuzeykent 37150, Samsun, Turkey; sgokmen@kastamonu.edu.tr; 4 Art de Huile—Aromatherapy, Zekeriyaköy 34450, Istanbul, Turkey; hulyakayhan@hotmail.com; 5 Department of Pharmacology and Toxicology, Faculty of Veterinary Medicine, University of Ondokuz Mayıs, Atakum 55270, Samsun, Turkey

**Keywords:** antibacterial effects, antibiofilm effect, essential oils, primary fibroblast cells

## Abstract

**Background**: Essential oils exhibit several biological activities such as antimicrobial, antioxidant, proliferative, and anti-inflammatory. This study was aimed at investigating the antimicrobial effects and cytotoxic activities of niaouli, palmarosa, and clove essential oils. **Methods**: Content analyses of these essential oils were carried out by gas chromatography–mass spectrometry. The antibacterial activity was screened against methicillin-resistant *S. aureus* ATCC 43300, *P. aeruginosa* ATCC 27853, *P. aeruginosa* PAO1, *S. aureus* ATCC 25923, and 44 isolates (22 *P. aeruginosa* isolates, 4 *S. aureus* isolates, and 18 *Staphylococcus* spp. isolates) obtained from dogs with previous wound infections who were included in the current study. The antimicrobial effects of essential oils were investigated using disk diffusion and minimum inhibition/bactericidal concentration methods. Additionally, the antibiofilm, protease, elastase, and gelatinase activities of the essential oils were evaluated. Different concentrations of each essential oil ranging from 10 to 1000 µg/mL were also analyzed in terms of cell viability by WST-8 assay in primary canine fibroblast cells. **Results:** The fibroblast cell viabilities of palmarosa, niaouli, and clove oils at a 1000 µg/mL concentration were 75.4%, 96.39%, and 75.34%, respectively. All the EOs were found to have bactericidal effects with MBCs/MICs of 0.015 to 0.5 µL/mL against *P. aeruginosa*, *Staphylococcus* isolates (*p* < 0.001). Palmarosa was found to have the largest inhibition zone diameter (20.5 ± 6.6, 16.4 ± 2.3) compared to other essential oils in the disk diffusion test against *Staphylococcus* spp. and *P. aeruginosa* (*p* < 0.001). But none of the EOs reduced protease, elastase, and gelatinase activities, which are some of the virulence properties of the tested bacteria. **Conclusions:** These results showed that palmarosa, niaouli, and clove essential oils act as potential antibacterial agents for dogs against *P. aeruginosa*, methicillin-resistant *S. aureus*, and *Staphylococcus* spp., without damaging the skin.

## 1. Introduction

Essential oils (EOs) are secondary plant metabolites that have been used for a very long time in traditional medicine because of their various properties. EOs and plant extracts are rich in a wide variety of metabolite compounds. EOs have gained an increasing amount of popularity recently as natural veterinary medicines. Some of these compounds show significant antimicrobial activity, which makes us think that they are promising candidates for new drugs to treat bacterial infections [1,2,3].

Essential oils have been widely investigated for their therapeutic potential in various pathologies. Essential oils and their components possess different pharmacological effects such as antibacterial, anti-inflammatory, antioxidant, and antitumor [4]. Essential oils have been reported in a number of studies to exhibit antimicrobial activity against a wide range of bacteria, including antibiotic-resistant strains [5]. The main components of *Cymbopogon martinii* (palmarosa) EO are geraniol, geranyl acetate, linalool, and β-caryophyllene. Palmarosa EO is rich in geraniol, an acyclic monoterpene, which provides the essential oil with significant antimicrobial and antioxidant properties. Palmarosa EO is generally recognized as safe by the United States Food and Drug Administration (FDA). Furthermore, palmarosa EO has antioxidant, repellent, and antifungal properties, as well as antibacterial properties [6]. *Melaleuca quinquenervia* (niaouli) EO contains high levels of monoterpenes and sesquiterpenes [7]. Nioli EO contains 60% 1,8 cineole (eucalyptol) and certain proportions of terpineol, β-Pinene, p-cymene, and linalool [8,9]. Niaouli EO is a natural preservative with effective antimicrobial activity against skin flora [10]. It was also reported that niaolo EO did not show cytotoxic effects in mammalian cells (WI-38 healthy human fibroblast cells) [11,12]. *Eugenia aromaticum* (clove) EO exhibits biological activities, such as antibacterial, antifungal, insecticidal, and antioxidant properties. The main components of clove EO are eugenol, eugenol acetate, and gallic acid. It is well known that both eugenol and clove essential oil phenolic compounds can inhibit a great number of Gram-negative and Gram-positive bacteria [13,14]. Clove EO is generally recognized as safe by the FDA and used in perfumes, cosmetics, and foods, as well as drugs [15].

*Pseudomonas aeruginosa* (*P. aeruginosa*) is a ubiquitously distributed opportunistic pathogen that is found in soil and water, as well as animal-, human-, and plant-host-associated environments. It also has the ability to infect both humans and animals, especially immunocompromised hosts [16]. It causes the most nosocomial infections worldwide and threatens public health. Due to antibiotic overuse and misuse in human and veterinary medicine, *P. aeruginosa* has an alarming number of multi-drug-resistant strains [17]. *P. aeruginosa* is a widely used model bacterium in all biological fields, especially to study virulence and bacterial social traits. *Staphylococcus aureus* (*S. aureus*), a commensal bacterium, is a major pathogen in people and animals. Opportunistic infections caused by *S. aureus* can induce toxic shock syndrome or staphylococcal food poisoning. Nosocomial infections with methicillin-resistant *S. aureus* (MRSA) are associated with greater mortality and human health care expenses. In veterinary medicine, *S. aureus* is a common commensal bacterium that causes abscesses, mastitis, pneumonia, and meningitis, inflicting economic losses in the livestock industry. *S. aureus* isolates from animals pose a public health danger, because staphylococci can be transmitted between cattle, pets, and humans. *S. aureus* strains, especially those isolated from companion animals, are mostly of a human origin and are transmitted between human owners and their animals [18].

Biofilms are clusters of bacteria that adhere to a surface and/or each other and are embedded in a self-produced matrix, typically acting as a “protective suit” for bacteria. Bacteria in the biofilm also become more resistant to chemicals such as antibiotics and disinfectants [19]. In controlling the biofilm, antibiofilm compounds can kill the planktonic cell or inhibit biofilm formation by living cells. In the lysis process, antibiofilm compounds can destabilize the matrix by sensitizing microbial cells within biofilms to antimicrobial and/or host defense mechanisms. In the attenuating approach, antibiofilm agents can neutralize virulence factors or affect processes involved in biofilm formation such as quorum sensing [20,21]. Compounds that could prevent biofilm formation and eliminate persistent biofilms are being studied; however, they seem insufficient [21,22]. There are studies investigating the potential inhibitory effects of various essential oils on the virulence properties of *P. aeruginosa* and *S. aureus* [23,24,25]. It is clear that new therapeutic options are needed to control biofilm-associated infections. Bacterial proteases (serine proteases, metalloproteases, etc.) are virulence factors that have key roles in cell physiology, replication, and survival and thus play an important role in pathogenesis, and they are also produced by pathogenic *S. aureus* and *P. aeruginosa* strains. Clinically, extracellular proteases are responsible for the destruction of host tissue and the degradation of host defense proteins such as IgA1 immunoglobin [26,27]. There are studies investigating the potential inhibitory effects of various essential oils on the virulence properties of *P. aeruginosa* and *S. aureus* [23,24,25,28]. Today, one of the strategies for solving the problem of antibiotic resistance is the use of alternative antimicrobials that are especially effective against biofilms, including the use of EOs obtained from plants [29].

Studies to determine the susceptibility of human isolates to various EOs in vitro are much more extensive than in vitro studies testing EOs against isolates of animal origin, and it is clear that more emphasis should be given to such studies in the field of veterinary medicine. It is also very important that these studies shed light on research confirming the in vivo usefulness of EOs, which have the potential to be used to treat animal infections. A subcutaneous fibroblast primary cell line was prepared to evaluate whether these EOs have a dermal toxic effect when applied topically. Fibroblasts at the wound site have a crucial role in tissue repair, as they control collagen deposition through proliferation and extracellular matrix protein synthesis [30].

The aim of this study was to investigate the in vitro the antibacterial effects of three selected EOs, *Cymbopogon martinii* (palmarosa), *Melaleuca viridiflora* (niaouli), and *Eugenia caryophyllus* (clove) essential oils, against *P. aeruginosa, S. aureus,* and *Staphylococcus* spp. pathogens that are frequently isolated from infections associated with antibiotic resistance.

## 2. Results

### 2.1. Essential Oil Composition

The chemical compositions of (%) the palmarosa, niaouli, and clove EOs are presented in Table 1.

### 2.2. Antibacterial Susceptibility

#### 2.2.1. Disk Diffusion Tests

The inhibition zone diameters formed by each EO at a constant 20 µL volume are given in Table 2. The diameters of the inhibition zones formed by the EOs on the reference strains are given in Table 3. Palmarosa oil exhibited the largest inhibition zone diameter of the Staphylococcus spp. and *P. aeruginosa* isolates compared to the other essential oils (*p* < 0.001). All three EOs were found to form zones that were close in diameter to the zone formed by antibiotics in the reference strains.

#### 2.2.2. MIC, MBC, and MBC/MIC Values of EOs

The MIC, MBC, and MBC/MIC values of each EO are summarized in Table 4. The MIC of clove EO was found to be 0.015 µL/mL on *Staphylococcus* spp. and *S. aureus* isolates and in the range of 0.015–0.5 µL/mL on *P. aeruginosa* isolates. While the MIC of palmarosa EO was in the range of 0.015–0.0625 µL/mL on *Staphylococcus* spp. and *S. aureus* isolates, it ranged from 0.015 to 0.5 µL/mL on *P. aeruginosa* isolates. The MIC of niaouli EO was 0.0039 µL/mL and 0.5 µL/mL on *P. aeruginosa* and *Staphylococcus* isolates, respectively. Based on the MBC/MIC values of the EOs, all EOs had bactericidal effects on all strains tested (*p* < 0.001).

#### 2.2.3. Biofilm Inhibition

**Figure 1:**
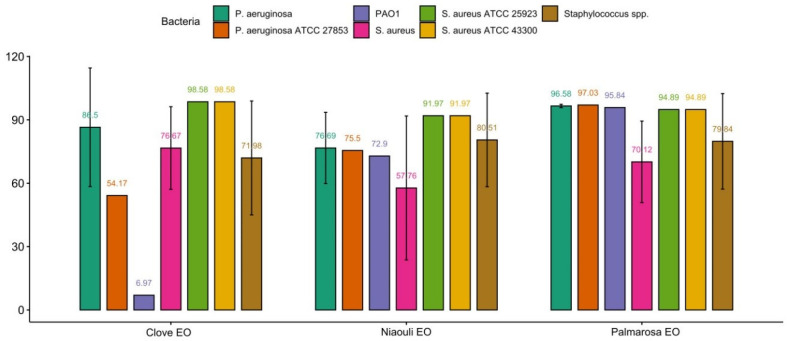
Percentages (%) of biofilm inhibition of EOs on clinical and reference strains.

The biofilm inhibition percentages of the EOs on strains are shown in Figure 1. It was determined that palmarosa achieved the highest biofilm inhibition in *Staphylococci* (78.07%) and *P. aeruginosa* (96.58%). Also, clove oil showed the highest biofilm inhibition (98.58%) in the *S. aureus* ATCC 25923 and *S. aureus* ATCC 43300 reference strains.

#### 2.2.4. Inhibition of Protease, Elastase, and Gelatinase

**Figure 2:**
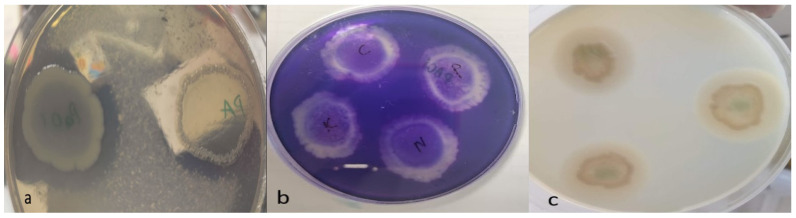
Bacterial enzyme activities. (**a**) Elastase activity, (**b**) Protease activity, (**c**) Gelatinase activity, PA: *P. aeruginosa* ATCC 27853, PAO1, C: Clove oil, P: Palmarosa oil, N: Niaouli oil, K: Control strain.

It was determined that none of the EOs reduced protease, elastase, and gelatinase activities, which are some of the virulence properties of the tested bacteria. However, the enzyme activities of the bacteria are given in Figure 2.

### 2.3. Cell Viability Test

**Figure 3:**
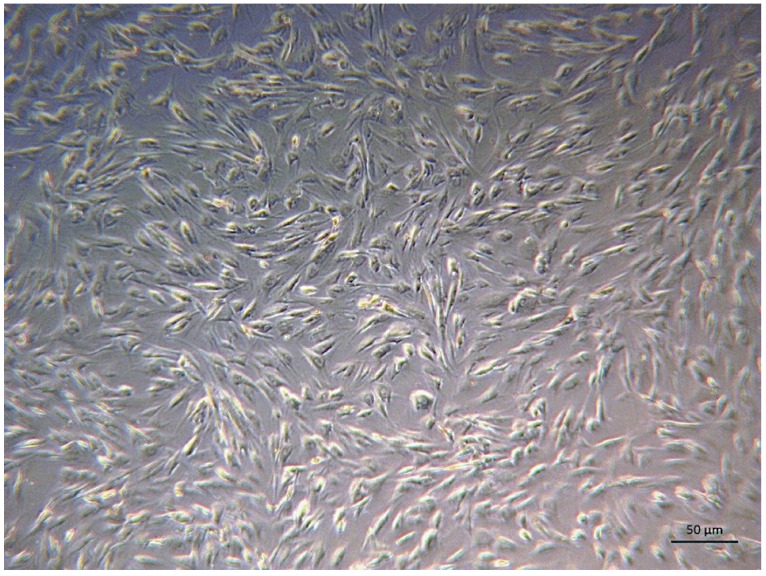
Canine primary fibroblast cells. Bar: 50 µm.

In this study, canine primary fibroblast cells were used to determine the viability of essential oils (Figure 3). The cell viabilities of palmarosa, niaouli, and clove EOs at different doses are given in Table 5. The half-maximal inhibitory concentration (IC_50_) value in fibroblast cells was not reached at concentrations between 75 and 1000 µg/mL of EOs. The fibroblast cell viabilities of palmarosa, niaouli, and clove oils at a 1000 µg/mL concentration were 75.4%, 96.39%, and 75.34%, respectively.

## 3. Discussion

Currently, when faced with the problem of antimicrobial resistance in both human and veterinary medicine, the use of various essential oils of a plant origin or their active components is considered as an alternative solution to this health problem. Volatile compounds obtained from plants, especially essential oils, are known to exhibit antimicrobial, fungicidal, and insecticidal activities with various phytochemicals they contain. The antibacterial effects of essential oils on various Gram-positive and Gram-negative bacteria have been investigated in numerous studies [31]. In our study, the antibacterial activities of three essential oils and their effects on various bacterial virulence factors were evaluated.

Palmarosa essential oil obtained from the *Cymbopogon martinii* plant is particularly rich in geraniol, and geraniol and geranylacetate compounds make up approximately 75–90% of the total essential oil and contain less linalool and β-caryophyllene. Terpenoids that are known to exhibit antimicrobial, antifungal, and antioxidant activities are found as essential oils in many plants (80%) [31]. In the current study, the major component of palmarosa oil was geraniol, and the geraniol content was determined to be 84.152%.

In this study, palmarosa EO formed a significant zone of inhibition on *S. aureus* and *Staphylococcus* species other than *S. aureus* at 24.75 and 20.8 mm, respectively, but the zone of inhibition on *P. aeruginosa* was smaller (16.8 mm). Gram-positive bacteria are known to be more sensitive to essential oils than Gram-negative bacteria. The weak antibacterial activity against Gram-negative bacteria has been explained, as the hydrophilic polysaccharide chains in the outer membrane structure of Gram-negative bacteria form a barrier against hydrophobic essential oils. Palmarosa oil impregnated on paper disks at a 100% concentration was reported to produce an inhibition zone of 22 mm for *S. aureus* in a disk diffusion test [32]. It was reported that palmarosa oil (200 µg/mL) was effective on *S. aureus* (the reference bacterial strain) but not on *P. aeruginosa* [33].

Here, it is seen that the zone diameters that we obtained are compatible with other studies. Palmarosa EO was reported to form smaller inhibition zones on *S. aureus* ATTC 25923, *P. aeruginosa* 27853, and PAO1 strains (which we also tested in this study) [34]. It is reported that palmarosa EO moderately inhibited *S. aureus* (14–18 mm), while *P. aeruginosa* was not inhibited, and these results were similar to those of Da Silva et al. [34,35,36,37]. It was thought that the different results obtained in the disk diffusion test may be due to differences in oil compositions and/or concentrations. Also, this difference can be explained by the fact that the compositions of the EOs vary according to the season, climate, and method of oil extraction [31,38]. The antimicrobial effect associated with geraniol terpenoid occurred due to a microorganism destroying the membrane structure and causing impaired cell integrity [22].

*Syzygium aromaticum* (clove) is widely used, as it has anti-inflammatory, antimicrobial, antithrombotic, antioxidant, antimutagenic, and anti-ulcerogenic properties. The high levels of eugenol found in clove essential oil are responsible for its potent biological and antimicrobial activities. It is well known that the phenolic compounds in both eugenol and clove essential oil can denature proteins and change their permeability by reacting with cell membrane phospholipids and inhibit a large number of Gram-negative and Gram-positive bacteria, as well as different yeast species [30,31,32,33,34,35,36,37,38,39]. It could be speculated that the bactericidal effect of the clove oil used in this study against *S. aureus* and *P. aeruginosa* is due to the 88% eugenol in its content.

Niaouli EO is rich in 1,8-cineole, and its composition varies greatly according to different geographical locations. Of the monoterpenoids, 1,8 cineole, pcymene, and terpineol are the compounds with the highest contents in its composition [40]. Synergistic activity has been shown between 1,8-cineole and chlorhexidine against *S. aureus* and MRSA, as well as *E. coli*, *K. pneumoniae, E. faecalis,* and *C. albicans*. The reason for this is explained by the fact that 1,8-cineol, like chlorhexidine, acts on the plasma membrane of bacteria [41]. Although the niaouli EOs used in the present study were ineffective against *P. aeruginosa* isolated from wound isolates, the bactericidal activity of niaouli against other isolates and all reference strains may be attributed to the 1,8 cinoel in its content.

Researchers consider the antimicrobial activity of extracts to be significant if the MIC value is 0.1 mg/mL or lower, moderate if 0.1 < MIC ≤ 0.625 mg/mL, and weak if MIC > 0.625 mg/mL [42]. Accordingly, it was observed that all EOs tested for their antibacterial activity in this study had a significant effect on staphylococci, while niaouli EO was not effective against any strain of *P. aeruginosa*. The effects of clove and palmarosa EOs ranged from effective to weak in the clinical isolates. The differences between the strains in terms of their virulence factors may cause this variability. Palmarosa EO has been found to be moderately effective against *P. aeruginosa* [33]. The MIC value of palmarosa oil on both *S. aureus* and *S. epidermidis* isolates is reported to be 5000 µg/mL. Both the method (agar dilution method) used to detect MIC values and the results were different from our results [43]. Nevertheless, MIC definitions diverge between publications, which is an additional problem concerning data comparison. It seems that palmarosa EO may be more effective on staphylococcus strains, although the MIC values vary. Alanazi et al. have reported that clove oil showed good antibacterial activity against MRSA in vitro with an MIC of 1.25 µL/mL and an MBC of 2.5 µL/mL [44]. In our study, it was also found to have a good antibacterial effect on the MRSA strain. Niaouli EO had a lower MIC value (0.0039 µL/µL) for *P. aeruginosa*, while it was 0.5 µL/µL for staphylococcal strains. Accordingly, it can be said that niaouli EO has no effect on *P. aeruginosa*. Moreover, in this study, it was determined that niaouli EO did not form an inhibition zone on *P. aeruginosa* culture when evaluated using the disk diffusion test.

Biofilm-producing bacteria are resistant to various harsh conditions and essential to an infection’s spread and persistence in a host. Therefore, controlling pathogenic biofilm formation is important in bacteria-related diseases [21]. In our study, palmarosa EOs inhibited the biofilm of staphylococci and *P. aeruginosa* more than other oils (78.07 and 96.58, respectively). Palmarosa EO has also been shown in other studies to cause biofilm inhibition in PAO1 and MRSA: 64% and 25.8%, respectively [34,35,36,37,38,39,40,41,42,43,44,45,46,47]. The higher geroniol ratio (84.15%) of palmarosa EO used in our study may be considered the reason why the MRSA and PAO1 strains had higher biofilm inhibition percentages of 94.89% and 95.84%, respectively. Merghnia et al. [48] found that niaouli EOs inhibited biofilm formation in clinical methicillin-resistant *S. aureus* (MRSA) strains in the range of 77.46 ± 1.91 to 90.81 ± 4.05 [48]. In our study, niaouli EO showed 91.97% biofilm inhibition in the reference MRSA strain. However, we found that the strongest biofilm inhibition against the reference MRSA strain was produced by clove EO (98.58%). The antibiofilm properties of essential oils may be due to cytoplasmic membrane modification by hydrophobic components [49], the membrane reactivity and diffusion rate of components, anti-quarum sensing properties, and inhibition of the transcription of flagellar genes [49,50]. Another study involving proteomic analysis of the efficacy of EO from Amomum villosum against MRSA *S. aureus* showed that its hydrophobicity and, consequently, bacterial adhesion to inert surfaces decreased and that this effect was dose- and temperature-dependent [51]. The antibiofilm activity of geraniol on MRSA *S. aureus* has been demonstrated by the inhibition of the main biofilm component, the polysaccharide intercellular adhesin (PIA) [52]. In our study, we reported that the highest biofilm inhibition we found in palmarosa oil may be due to the high geraniol level in its content.

The biofilm prevention effect of palmarosa EO against *P. aeruginosa* and of niaouli EO against MRSA suggests that combining their formulations may be appropriate for clinical use in the veterinary field.

In the last two decades, numerous experimental studies have been conducted on antibiofilm- and virulence-factor-reducing EOs and EOCs (essential oil compounds) [19]. EOs are known to contain biologically active compounds (such as inhibiting virulence factors) that may contribute to the discovery and development of new antimicrobial therapeutics. It was reported that clove bud oil significantly decreased the activity of some virulence factors, such as elastase A and elastase B of *P. aeruginosa*. Eugenol, an important component of clove EO, has been proven to be effective in combating various pathogens such as *S. typhi, P. mirabilis, E. coli*, *S. aureus*, and *P. aeuriginosa* by changing the integrity of cell membranes [46]. It was also reported that palmarosa EO decreased elastase activity by 64% [34]. Although all clinical isolates exhibited protease, elastase, and gelatinase activities in our study, none of the essential oils tested caused these isolates to decrease in protease, gelatinase, and elastase activities. It was thought that this situation might depend on the dose of essential oil used, the composition of the active ingredients contained in the essential oils, and the test method.

The antibacterial action of EOs varies based on the oil and microorganism. A great deal of variation has been observed between bacterial species and between strains of a given species. A comparison of results from different studies is not easy due to the variability in the composition of the same EOs, as well as the methods used to determine the susceptibility of the agents. Although there are some differences, it is possible to judge the suitability of some compounds as promising candidates for treating microbial diseases [1].

In this study, palmarosa, niaouli, and clove EOs showed less than 50% inhibition in the canine primary fibroblast cell line at the highest concentrations (1000 µg/mL), falling within the linear range of the initial-end ratios. We have clearly demonstrated that palmarosa, niaouli, and clove EOs can show antibacterial activity against these bacteria. Although further clinical studies are required, these findings suggest that palmarosa, niaouli, and clove EOs may be used in wound infections in dogs as a antibacterial agent.

## 4. Materials and Methods

### 4.1. Essential Oils

Niaouli (*Melaleuca viridiflora*), palmarosa (*Cymbopogon martinii*), and clove (*Eugenia caryophyllus*) EOs were supplied by Art de Huile (Istanbul, Turkey).

### 4.2. Gas Chromatography–Mass Spectrometry (GC-MS)

The supplier’s essential oil content analysis is provided below. The GC analysis of the EOs was performed on an Agilent 7890A/5975C (Agilent Technologies, Santa Clara, CA, USA) with an FID. A capillary column (50 m × 0.25 mm i.d., 0.2 μm film thickness, CPWAX 52 CB) was used to separate the EO components. Analysis was carried out using helium as a carrier gas at a flow rate of 2 mL/min. Mass spectrometry was performed at scan mass range (*m*/*z*) from 30 to 500 amu. An oil solution was prepared by dissolving 100 μL of the EOs (each separately) in 1 mL of hexane prior to GC analysis.

### 4.3. Bacterial Strains

A total of 44 bacterial isolates (22 *P. aeruginosa* isolates, 4 *S. aureus* isolates, and 18 *Staphylococcus* spp. isolates) isolated from dogs with wound infections that were previously treated and preserved in the culture collection of Ondokuz Mayıs University, Veterinary Microbiology Department, were used. In addition, MRSA ATCC 43300 (Refik Saydam Hıfzısıhha (Hygiene) Institute, Ankara, Turkey), *P. aeruginosa* ATCC 27853 (Refik Saydam Hıfzısıhha (Hygiene) Institute, Ankara, Turkey), *P. aeruginosa* 01 (PAO1) (Refik Saydam Hıfzısıhha (Hygiene) Institute, Ankara, Turkey), and *S. aureus* ATCC 25923 (Refik Saydam Hıfzısıhha (Hygiene) Institute, Ankara, Turkey) strains were used as control strains.

### 4.4. Determination of Antibacterial Activities of EOs by Disk Diffusion Method

Antimicrobial activity tests were performed using the disk diffusion and broth dilution method, following the Clinical and Laboratory Standards Institute (CLSI) guidelines. The paper disk diffusion method was used to screen the essential oils’ antibacterial activity on the strains. The density of 0.5 McFarland bacterial suspensions was prepared in saline, and each bacterial suspension (100 µL) was spread on Mueller–Hinton agar. Blank paper disks (6 mm) were saturated with 20 µL of each EO and placed onto Mueller–Hinton agar plates. Amoxicillin/clavulanic acid (30 µg) and gentamycin (5 µg) disks were also used as controls. The plates were incubated at 37 °C for 24 h. The diameter of the clear zone surrounding the disks was measured.

### 4.5. Determination of Minimal Inhibitory Concentrations (MICs) and Minimum Bactericidal Concentrations (MBCs) of EOs by Broth Microdilution Method

Each EO’s bacterial MIC and MBC were determined using the microdilution method according to the Clinical and Laboratory Standards Institute guidelines. A homogenous solution was made by mixing equal quantities of the EOs (each separately) and dimethyl sulfoxide (DMSO, 10%). Serial two-fold dilutions of EO (50%) in Mueller–Hinton Broth were prepared in a 96-well microplate (90 µL/well). Starting from 50% dilution, eight steps of two-fold dilution were completed. Bacterial suspensions (10 µL) were added to each well at a final concentration of 5 × 10^5^ CFU/mL. Microplates were incubated at 37 °C for 24 h. The MIC was defined as the lowest concentration that inhibited visible growth. The MBC was determined by subculturing 100 µL of each negative test tube onto Tryptic Soy Agar (TSA) plates. The MBC was defined as the lowest concentration that resulted in a negative subculture or the presence of only one colony after incubation. The MBC/MIC ratio was used to determine the bacteriostatic or bactericidal property of each EO (≥4 bacteriostatic, ≤4 bactericidal, and =1 absolute bactericidal effect).

### 4.6. Inhibition of Biofilm Formation

The biofilm inhibition activities of the EOs were determined using crystal violet stain. Strains were grown in Luria–Bertani Broth overnight. The overnight culture was diluted 1:100 with a fresh medium, and 100 µL was added to the wells. The same strains were also mixed with the EO to be tested in such a way that their final concentration was similar to their sub-MICs and placed in the wells. At the end of the incubation (37 °C, 24 h), the microplates were washed with water and crystal violet (200 µL, 0.1) was added to each well. After incubation (30 min at room temperature), the plates were washed and allowed to dry. Then, acetic acid (30%, 125 µL) was added, and the crystal violet was allowed to dissolve for 15 min, and the solubilized crystal violet was transferred to a clean microplate well. The absorbance was read at 595 nm using the a microplate spectrophotometer (ThermoFisher Scientific, Waltham, MA, USA). The EO (inhibitor)-mediated reduction in biofilm formation was evaluated in comparison with the oil-free control. The standard antibiotics amoxicillin (2 µg/mL) and gentamycin (10 µg/mL) were used as positive controls, and an EO-free medium was used as a negative control. The percent biofilm inhibition was calculated as follows:

Biofilm inhibition (%) = (Control OD595 nm − Test OD595 nm/Control OD595 nm) × 100

### 4.7. Inhibition of Protease Activity

Protease tests were performed according to Vijayaraghavan and Vincent with some modifications [47]. Isolates’ overnight fresh broth cultures with or without EOs were centrifuged for 10 min at 10,000 rpm to obtain the enzyme. A loopful of supernatant was inoculated on Skimmed Milk Agar (SMA) in dots and incubated at 28 °C for 24 h. The culture supernatant mixed with EOs at their sub-MICs was also inoculated into the SMA plates simultaneously. A crystal violet solution (0.015%, 5 mL) was added to agar plates and incubated at room temperature for 20–30 min. The formation of a clear zone was evaluated as positive in terms of protease activity, and the absence of a zone was evaluated as negative or inhibited protease.

### 4.8. Inhibition of Elastase Activity

The elastolytic activities of culture supernatants alone and in the presence of EOs were tested by modifying the method used by Stewart [30]. Isolates and reference strains were inoculated on elastin-TSA (3 g/L) plates and incubated at 37 °C for 24 h. The zone formation around the spot-inoculated area was evaluated as positive for elastase.

### 4.9. Inhibition of Gelatinase Activity

Gelatin hydrolysis tests were performed according to dela Cruz and Torres [34]. A heavy inoculum of 18 to 24 h old test bacteria were spotted onto agar nutrient gelatin agar medium (8 g/L) and incubated at 37 °C for 24 h. At the end of the incubation, a saturated ammonium sulfate solution (38.05 g/50 mL) was poured into the Petri dish and left for 5–10 min so that the zone diameters became visible. Gelatin hydrolysis was determined by the observation of clear areas around the colonies [53].

### 4.10. Primary Canine Fibroblast Culture

We obtained fibroblasts from an intact piece of skin taken 20 min earlier from a dog who was euthanized due to a motor vehicle accident and admitted to Ondokuz Mayıs University Faculty of Veterinary Medicine Animal Hospital’s Small Animal Clinic. Ethics Committee approval was not required. We modified Aguilera-Rojas et al.’s technique to produce a primary fibroblast cell culture [35]. Briefly, the tissue sample was mixed into a Dulbecco’s Modified Eagle Medium (DMEM, Gibco, Waltham, MA, USA) solution containing sterile penicillin/streptomycin (15140122, Gibco, Waltham, MA, USA) and then placed in a Petri dish. The dermis was separated from the epidermis using sterile forceps and a scalpel. Cells isolated from canine skin tissue were morphologically identified as fibroblasts, because they have long spindle-like cytoplasmic extensions and oval nuclei. The tissue was cut into small pieces in the DMEM solution. After this process, the tissue was incubated at 37 °C with 5% CO_2_. Fibroblasts were seen on the tenth day, small tissue fragments were removed, and the medium was altered. After fibroblast proliferation was observed for 12 days, these cells were passaged. During the passage process, the medium was removed, and 3 mL of trypsin (59418c, Sigma-Aldrich, St. Louis, MO, USA) solution was added to the Petri dish and incubated at 37 °C for 5 min. After incubation, 3 mL of medium was added and pipetted. The obtained medium was placed into a falcon tube and centrifuged at 1500 rpm for 5 min. The supernatant was discarded, and the remaining residue was re-suspended in 1 mL of medium and mixed. Cells were added to the medium containing 20% fetal bovine serum (1050064, Gibco, Waltham, MA, USA). It was determined whether the cell density reached 70–80%. When the desired cell density was reached, the cells were subcultured again. After separating cells with trypsin and centrifugation, the supernatant was discarded, and 1 mL of medium was pipetted over the remaining residue. Ten microliters of the medium was combined with ten microliters of trypan blue solution. Cells were counted using TC20 Automated cell counter (Bio-Rad, Segrate, Italy).

### 4.11. WST-8 Test

For the cytotoxicity test, cells were distributed in a 96-well plate with 10,000 cells per well. Niaouli, palmarosa, and clove EOs were used. Stock solutions were prepared by dissolving the essential oils in DMSO (0.2%) to 1000 µg/mL as a master stock. For niaouli and clove, 2.3 µL/mL, and for palmarosa, 2 µL/mL were precipitated in the medium. Sub-dilutions were made from the stocks for each oil. For this purpose, the amount of undiluted essential oils in the medium used in the preparation of sub-dilutions was 2 and 2.3 µL/mL for 1000 µg/mL; 1 and 1.15 µL/mL for 500 µg/mL; 0.5 and 0.575 µL/mL for 250 µg/mL; 0.2 and 0.23 µL/mL for 100 µg/mL; and 0.15 and 0.1725 µL/mL for 75 µg/mL of palmarosa and niaouli or clove EO, respectively. Then, 100 µL of the samples prepared at the indicated doses was added to each well. Finally, 20 µL serum was added to each well and incubated at 37 °C with 5% CO_2_ for 24 h. After 24 h of incubation, the solution in each well was removed, and then, 100 µL of medium was added to each well. Following this, 10 µL of WST-8 solution (ab228554 Cell Counting Kit 8, Abcam, Cambridge, MA, USA) was added to them and incubated (at 37 °C, 5% CO_2_) for 1 h. It was read at a 460 nm absorbance value in the Elisa Reader (Infinite F50, Tecan, Männedorf, Switzerland). As a result, the cytotoxicity of the EO was determined. The cell viability results obtained from the WST-8 test are shown in Table 1. DMSO was used for vehicle control. Results were expressed as a percentage of cell viability compared to the control.

Each test was performed in triplicates, and the results were evaluated.

### 4.12. Statistical Analysis

Data analysis was performed in RStudio 2022.07.2+576 (RStudio, PBC, Boston, MA, USA). A Kruskal–Wallis test was used to determine whether there is a statistically significant difference between the medians of the three EO groups. The α level was taken as 0.05. Dunn’s test was applied for pairwise comparisons between each independent group after the statistically significant groups were determined, and all the p-values calculated within the pairwise comparisons were adjusted according to the Holm method. Effect sizes were evaluated according to Cohen [36].

## Figures and Tables

**Table 1:** Chemical compositions of EOs.

Clove EO	Palmarosa EO	Niaouli EO
Eugenol (88.013%)	Geraniol (84.152%)	1,8-Cineole (70.382%)
Eugenol acetate (9.488%)	Gerenayl acetate (8.343%)	α-Terpineol (6.265%)
Caryophyllene (1.878%)	Linalool (3.090%)	α-Pinene (6.686%)
1-octanol (0.321%)	Cis Beta Ocimene (0.972%)	dl-Limonene (5.413%)
α-capaene (0.215%)	trans-Caryophyllene (0.773%)	Veridiflorol (2.053%)
Methyl salicylate (0.078%)	Geranial (0.768%)	β-pinene (1.847%)
	Geranyl isobutyrate/Geranyl Hexanoate (0.574%)	α-Terpinene (1.056%)
	trans-Farnesol (0.403%)	γ-Terpinene (1.037%)
	β Ocimene (0.280%)	trans-Caryophyllene (0.903%)

**Table 2:** Inhibition zone diameters (mm) of EOs on isolates.

Essential Oil	*Staphylococcus* spp.	*S. aureus*	*P. aeruginosa*
Clove	17 ± 4.6 ^a^	14.25 ± 3.5	7 ± 5.4 ^a^
Palmarosa	20.5 ± 6.6 ^b^	24.75 ± 9.5	16.4 ± 2.3 ^b^
Niaouli	19.5 ± 4.4 ^a,b^	23.25 ± 4.5	Resistant ^c^

^a, b, c^ Different superscript letters in the same column indicate statistical significance.

**Table 3:** Inhibition zone diameters (mm) of EOs on reference strain.

*S. aureus* ATCC 25923					*S. aureus* ATCC 43300					*P. aeruginosa* ATCC 27853				PAO1			
C	P	N	AMC	GN	C	P	N	AMC	GN	C	P	N	GN	C	P	N	GN
17	18	18	28	16	16	15	15	16	15	15	12	18	16	13	14	16	15

C: clove oil; P: palmarosa oil; N: niaouli oil; AMC: amoxicillin–clavulanic acid; GN: gentamycin.

**Table 4:** MIC, MBC, and MBC/MIC values of EOs on strains (µL/µL).

Essential Oil	*Staphylococci*	*P. aeruginosa*	*S. aureus* ATCC 25923	*S. aureus* ATCC 43300	*P. aeruginosa* ATCC 27853	PAO1
Clove	0.015 ^a^	0.015–0.5 ^a^	0.015	0.015	0.015	0.015
Palmarosa	0.015–0.0625 ^b^	0.015–0.5 ^b,c^	0.015	0.015	0.5	0.5
Niaouli	0.0039 ^b,c^	0.5 ^c^	0.0039	0.0039	0.5	0.5

^a, b, c^ Different superscript letters in the same column indicate statistical significance at *p* < 0.001.

**Table 5:** Cell viabilities of palmarosa, niaouli, and clove EOs.

Concentration	Cell Viability %			
	Palmarosa	Niaouli	Clove	DMSO
1000 µg/mL	75.4 ^a^	96.3 ^b^	75.3 ^a^	103.9 ^b,c^
500 µg/mL	82.8 ^a^	107.0	111.1	106.6
250 µg/mL	82.9 ^a^	103.1	106.5	102.7
100 µg/mL	83.8 ^a^	101.6	100.8	101.5
75 µg/mL	81.9 ^a^	100.9	96.2	102.8

^a, b, c^ Different superscript letters in the same line indicate statistical significance at *p* < 0.001.

## Data Availability

Data are contained within the article.
